# Childhood Correlates of Blood Lead Levels in Mumbai and Delhi

**DOI:** 10.1289/ehp.8399

**Published:** 2005-10-20

**Authors:** Nitin B. Jain, Howard Hu

**Affiliations:** 1 Channing Laboratory, Brigham and Women’s Hospital and Harvard Medical School, Boston, Massachusetts, USA; 2 Department of Environmental Health, Harvard School of Public Health, Boston, Massachusetts, USA; 3 Department of Environmental Health and Engineering, Sri Ramachandra Medical College and Research Institute, Chennai, Tamil Nadu, India

**Keywords:** children, India, lead

## Abstract

**Background:**

Lead exposure has previously been associated with intellectual impairment in children in a number of international studies. In India, it has been reported that nearly half of the children have elevated blood lead levels (BLLs). However, little is known about risk factors for these elevated BLLs.

**Methods:**

We conducted a retrospective cross-sectional analysis of data from the Indian National Family Health Survey, a population-based study conducted in 1998–1999. We assessed potential correlates of BLLs in 1,081 children who were < 3 years of age and living in Mumbai or Delhi, India. We examined factors such as age, sex, religion, caste, mother’s education, standard of living, breast-feeding, and weight/height percentile.

**Results:**

Most children (76%) had BLLs between 5 and 20 μg/dL. Age, standard of living, weight/height percentile, and total number of children ever born to the mother were significantly associated with BLLs (log transformed) in multivariate regression models. Compared with children ≤3 months of age, children 4–11 and 12–23 month of age had 84 and 146% higher BLLs, respectively (*p* < 0.001). A low standard of living correlated with a 32.3% increase in BLLs (*p* = 0.02). Children greater than the 95th percentile for their weight/height had 31% (*p* = 0.03) higher BLLs compared with those who were below the 5th percentile for their weight/height.

**Conclusions:**

Our study found various factors correlated with elevated BLLs in children. The correlation between greater than the 95th percentile weight/height and higher BLL may reflect an impact of lead exposure on body habitus. Our study may help in targeting susceptible populations and identifying correctable factors for elevated BLLs in Mumbai and Delhi.

Environmental lead exposure occurs from burning of fossil fuels, mining, and manufacturing and from drinking water where lead pipes are used. Exposure at home may occur through ingestion of old leaded paint, and pigments and glazes used in pottery [Agency for Toxic Substances and Disease Registry (ATSDR) 2000]. Some health care products and folk remedies are also known to contain lead (ATSDR 2004). The adverse health effects of lead pollution are known to be more pronounced in vulnerable populations such as children and members of socioeconomically disadvantaged communities (Rogan and Ware 2003). Evidence associating elevated lead levels in the body with decline in early cognitive function (Bellinger et al. 1987; McMichael et al. 1988; Needleman et al. 1979), delayed mental and physical development, hearing impairments (U.S. Consumer Product Safety Commission 2005), and intellectual impairment even at low levels (Canfield et al. 2003) has been presented. We have also reported the association of elevated lead levels in the body with varying severity of anemia in children (Jain et al. 2005).

After measures to control lead pollution were implemented in the United States, beginning in 1970, blood lead levels (BLLs) in children have declined by > 80% (ATSDR 2000). Conversely, lead pollution remains a public health concern in developing countries such as India. Previous studies based on regional data have estimated that more than half of the children in India have BLLs ≥10 μg/dL (George Foundation 1999; Kaul 1999; Patel et al. 2001), which is the Centers for Disease Control and Prevention (CDC) definition of elevated BLL in children (CDC 2000). However, only a few studies with relatively small sample sizes have attempted to investigate factors associated with BLLs in Indian children (Kalra et al. 2003; Patel et al. 2001).

The 1998–1999 National Family Health Survey (NFHS 2000) is the first to provide information on BLLs in children < 3 years of age in two major Indian metropolitan cities (Mumbai and Delhi). In agreement with earlier studies (Kaul 1999; Patel et al. 2001), the results of the NFHS indicated that approximately 50% of children in Mumbai and 45% in Delhi had BLLs ≥10 μg/dL [Centre for Operations Research and Training (CORT) and International Institute for Population Sciences (IIPS) 2000; IIPS and Opinion Research Corporation (ORC) Macro 2000b]. Information on proportion of children in different BLL categories across few sociodemographic and other variables was also presented. However, multivariable analysis of these factors was not performed, and other known predictors of BLL were not examined or controlled for. Also, the contribution of each factor toward predicting BLL was not described. We therefore assessed various sociodemographic, economic, and other factors that may be correlated with BLL in children < 3 years of age from a population-based survey conducted with a systematic sampling design in Mumbai and Delhi.

## Materials and Methods

### Database description and sampling design.

The NFHS was conducted with support from the U.S. Agency for International Development (USAID) and United Nations Children’s Fund (UNICEF) (NFHS 2000). The survey was carried out to assess the population health and nutrition in India. The NFHS was a household sample survey with an overall sample size of approximately 90,000 ever-married women, 15–49 years of age, living in 92,486 households, from all 26 states of India (bIIPS and ORC Macro 2000b). These women completed a structured interview to provide information regarding their family and living conditions. BLLs were obtained in the NFHS survey only in the metropolitan cities of Mumbai and Delhi, which may not represent the entire nation. Hence, further discussion is limited to these areas.

In Delhi, a three-stage stratified sampling design was employed for urban and rural areas separately (aIIPS and ORC Macro 2000a). The procedure started with either a ward (urban areas) or a village (rural areas) as the initial sampling strata, followed by selection of second-level sampling units based on probability proportional to size, and finally resulted in the selection of households using systematic sampling methodology. Of the 3,063 households selected in Delhi, 91% of eligible women completed the survey. In Mumbai, the census list of wards was used to determine primary sampling units based on their respective share of the population (CORT and IIPS 2000). A block of 150–200 households was selected per primary sampling unit using the probability proportional to size methodology. From these blocks, households to be interviewed were selected with equal probability using a systematic sampling procedure. There was a 91% overall response rate in Mumbai, resulting in 2,010 eligible women being interviewed. Further details on sampling design and procedures can be obtained from the NFHS website (CORT and IIPS 2000;a IIPS and ORC Macro 2000a,  IIPS and ORC Macro 2000b).

Women were also consented for inclusion of their children < 3 years of age in the NFHS survey. Permission for blood samples of children to measure BLL and hemoglobin was obtained. Database validation was performed by field editors and field supervisors and was further verified during processing. The present study complied with all applicable requirements of the United States and/or international regulations and was approved by the institutional review board of Brigham and Women’s Hospital.

### Sample selection.

The database included 1,082 children with information on BLLs. One child with a reported BLL but coded as being dead in the database was excluded. Of the remaining 1,081 children in our analysis, only 105 had siblings.

### Measurement of exposure.

The child’s hand or foot was first thoroughly washed with soap and water, and two or three drops of blood from a finger prick (or heel prick in the case of infants) was mixed with a treatment reagent and then transferred to a sensor by a pipette. The sensor was then introduced into a LeadCare analyzer (LeadCare Inc., Chelmsford, MA, USA), which displayed the results. Free treatment option was offered for any child with a lead level ≥45 μg/dL.

The reliability and accuracy of the LeadCare instrument have been verified in laboratory conditions as well as in the setting of highly contaminated environments through the analysis of split samples and comparisons with graphite furnace atomic absorption spectrometry (Counter et al. 1998; Pineau et al. 2002). Verification studies have also been conducted with similar results in India [All India Institute of Medical Sciences (AIIMS) 1999; CORT and IIPS 2000; IIPS and ORC Macro 2000b].

### Sociodemographic and other variables.

The NFHS calculated a standard of living index (bIIPS and ORC Macro 2000b) by assigning an index score to each household based on characteristics such as house type, toilet facility, source of lighting, main fuel for cooking, source of drinking water, separate room for cooking, house ownership, and ownership of agricultural land, irrigated land, livestock, and durable goods. The index scores ranged from 0–14 for low, 15–24 for intermediate, and 25–67 for high standard of living. Weight/height percentile was calculated by NFHS using the CDC standard-deviation–derived growth reference curves (Demographic and Health Surveys 2004). If the height or weight were outside the acceptable range for calculation of height/weight percentile, then it was coded as missing (*n* = 78) by the NFHS. Hemoglobin was measured in the field by using a portable HemoCue system (HemoCue Inc., Angelholm, Sweden). Anemia was defined according to the World Health Organization’s criteria of hemoglobin < 11.0 g/dL in children (WHO/United Nations/UNICEF 2001). Hemoglobin value was replaced as missing for one child with a reported hemoglobin of 0.8 g/dL.

### Statistical analysis.

**Figure 1:**
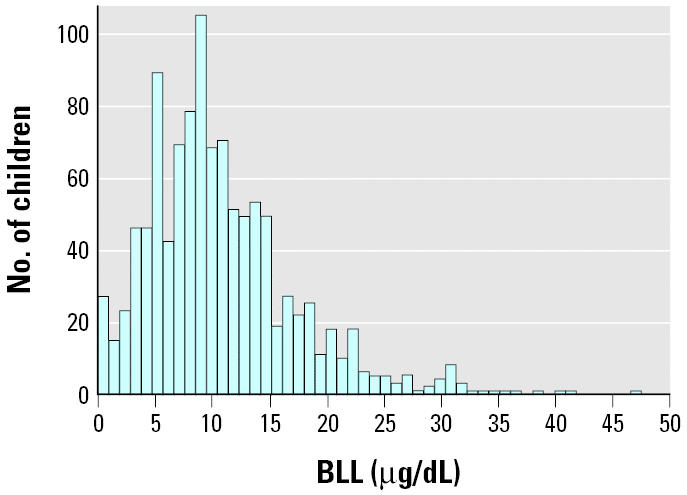
BLLs (*n* = 1,078) in children < 3 years of age in Mumbai and Delhi, India.

Mean BLLs were calculated across variables of interest. Analysis of variance tests were performed to assess the statistical significance of these variables in predicting BLL after a logarithmic scale conversion (because the distribution of BLL was skewed; Figure 1). Variables significant at the 0.10 level were assessed in multivariate linear regression models, after including age, sex, mother’s education, and standard of living index in the base model. Because 105 children in our study had siblings, a sensitivity analysis was done by excluding these children from the final multivariate analysis. The correlation of BLLs between the two youngest children of the same mother was also determined. Statistical analyses were conducted using Intercooled STATA for Windows (version 8.0; StataCorp, College Station, TX, USA) and SAS for Windows (version 8.02; SAS Institute Inc., Cary, NC, USA).

## Results

**Figure 2:**
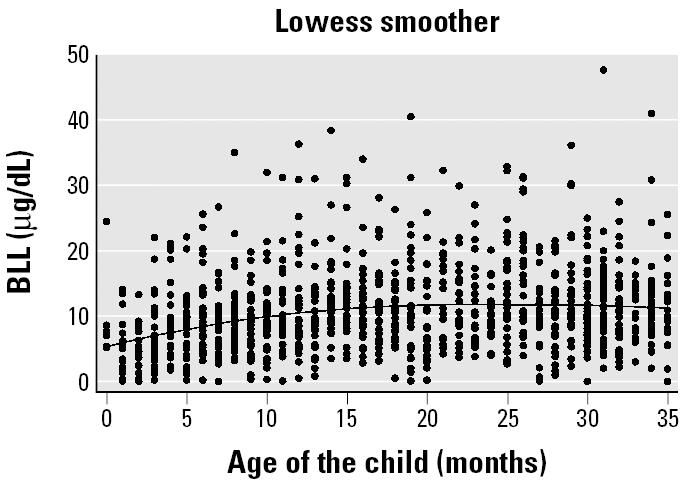
Scatter plot and smoothed line of BLLs (*n* = 1,078) by age of the child for children < 3 years of age in Mumbai and Delhi, India (bandwidth = 0.8).

Most children in our study had BLLs between 5 and 20 μg/dL (76%). BLLs increased significantly with increasing age in our study population (Table 1, Figure 2). Although few children (4.5%) had a low standard of living, BLLs were significantly higher [mean ± SD = 13.0 ± 6.7 μg/dL] in this group compared with children who had an intermediate (BLL mean ± SD = 11.2 ± 6.5 μg/dL) or high (BLL mean = 10.2 ± 6.5 μg/dL) standard of living. Children whose weight/height was greater than the 95th percentile also had significantly higher mean BLLs (12.0 μg/dL) compared with those with weight/height below the 5th percentile (10.9 μg/dL) or in the 5th–95th percentile range (10.8 μg/dL) (Table 1).

In the multivariate regression models, after controlling for age, sex, mother’s education, and standard of living index, the variables significantly associated with BLL (when converted to a logarithmic scale) were weight/height percentile and total number of children ever born to the mother (Table 2). Although duration of breast-feeding was significantly associated with BLL in the univariate analysis, its effect was largely accounted for by age in the multivariate regression analysis. When dichotomized into whether the child was currently breast-feeding or not, the variable was not significantly associated with BLL. Also, after adjusting for standard of living index, other possible indicators of socioeconomic status were not significantly associated with BLL. The final multivariate model was also analyzed by categorizing BLL into < 10 and ≥10 μg/dL (Table 3).

**Figure 3:**
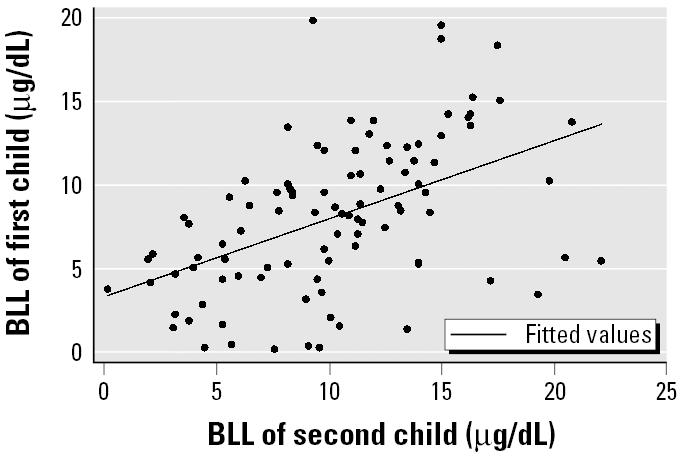
Scatter plot representing correlation in BLLs of children with siblings. Six children with BLLs ≥ 25 μg/dL were excluded to obtain a better representation on the graph; however, the graph was similar if the six children were included. Correlation coefficient = 0.48 (*p* < 0.0001); *n* for the correlation coefficient is 103 (not 105) because two children had two siblings in the cohort.

A positive correlation (*r* = 0.48; *p* < 0.0001) was observed between BLLs of children with siblings in the cohort (Figure 3). A sensitivity analysis including only the youngest child from a household was performed to test possible clustering of children within the same household. Results obtained from this analysis (*n* = 856 for multivariate model) were similar to ones presented in Table 2.

## Discussion

Lead pollution is a public health issue of concern in India, and elevated BLLs have been widely reported in Indian children. However, only a few studies have investigated factors that may be correlated with BLLs in Indian children. Lack of data on lead measurements and an adequately designed cohort with sufficient sample size has precluded such an assessment in the past. We used data from the NFHS on 1,081 children in Mumbai and Delhi to demonstrate various factors correlated with BLLs. After adjusting for sex and mother’s education, the variables significantly associated with higher BLLs were increasing age, lower standard of living index, greater than the 95th weight/height percentile, and higher total number of children ever born to the mother.

The high proportion of children with BLLs ≥10 μg/dL in our study may be exposed to lead from a variety of sources. However, leaded gasoline, which was still being used at the time of data collection, was likely to be a major contributor. The government of India has recently phased out the use of leaded gasoline, which may help to reduce lead exposures in India.

Rabinowitz et al. (1985) enrolled 249 newborns in Boston to determine correlates of BLLs. They reported that the strength of association between environmental lead and BLL increased with age, but demographic variables such as race, maternal age and education, and gender did not predict BLLs. In a study in 1- to 5-year-old Mexican children (*n* = 371), geometric means of BLLs did not show significant variation by age, sex, occupation, and education of mother. BLLs were associated only with the use of lead-glazed pottery dishes in the household and the habit of biting colored pencils among children (Lopez-Carrillo et al. 1996). However, Sargent et al. (1995) in their study of children from birth to 4 years of age in Massachusetts reported various sociodemographic and housing characteristics to be significant independent predictors of lead poisoning (defined as BLL ≥25 μg/dL). Potula and Hu (1996) assessed occupational and lifestyle determinants of BLL in 129 adult men in Madras, India. They reported that a nonvegetarian diet and job category were significant predictors of BLL. Other studies on the extent and sources of lead pollution in India have also been performed (Awasthi et al. 1996; Chatterjee and Banerjee 1999; Dwivedi and Dey 2002; Friberg and Vahter 1983; Gogte et al. 1991; Tripathi et al. 2001).

In our study, age was the strongest predictor of BLL, such that mean BLLs rapidly increased in children 4 months of age up to 23 months and then remained relatively steady (minor decline in the multivariate models). Although we did not have data on BLLs beyond 35 months of age, when the relationship of age and BLL was assessed more closely, the peak was observed at 26 months of age (mean BLL = 14.4 μg/dL; data not shown). A similar relationship of age with BLL has also been reported in population surveys conducted in the United States. Data on children 12–35 months of age from the 1988–1994 Third National Health and Nutrition Examination Survey showed peak BLLs in children 18–20 months of age (geometric mean = 4.1 μg/dL) and 24–26 months (geometric mean = 3.7 μg/dL) (Homa DM, Brown MJ, personal communication). Children who were 12–14 months of age had geometric mean BLLs of 3.1 μg/dL. BLLs showed a minor decline in children 27–35 months of age to 3.3 μg/dL at 33–35 months of age. Other studies also agree with these findings (Baghurst et al. 1992, 1999; Billick et al. 1979; Brody et al. 1994). A likely explanation for our results of increasing lead levels with age and a final plateau (Figure 2) is that children get more mobile as they grow older. This exposes them to lead from various sources such as paint, soil, and food. Research on activity patterns of children with age may help to determine whether this phenomenon is a major risk factor for lead exposure.

We found the standard of living index, which reflects socioeconomic status, to be significantly correlated with BLL in our study. Quinn et al. (1985) performed a study in the United Kingdom and also found social class to be independently negatively correlated with BLL in children. Moore et al. (1982) reported social class to be significantly associated with BLL in mothers and their 6-week-old children and explained the variation on the basis of correlation between the level of lead in water and social class. In our cohort, it is likely that the standard of living index was a proxy for environmental exposures such as lead from dust or drinking water, proximity to vehicular traffic, occupational lead exposures, lead from pottery and utensils, lower standards of hygiene, and possibly also the use of ayurvedic medicines containing lead (Saper et al. 2004). Similarly, we also found that children with mothers who had a greater number of children ever born had significantly higher BLLs. The total number of children in a family is a possible surrogate for socioeconomic status in our cohort. In fact, our data showed that 71.7% of families with a high standard of living index had ≤2 children, whereas only 52.8% and 46.9% families in the intermediate and low standard of living index had ≤2 children, respectively (data not shown). However, several socioeconomic factors not accounted for by the standard of living index may be responsible for higher BLLs in these children.

Our finding that a weight:height ratio greater than the 95th percentile is associated with higher BLLs is of unclear significance. It is possible that weight:height ratio is a proxy for otherwise-unmeasured environmental and/or biologic determinants of lead exposure. Conversely, it is possible that this relationship reflects an impact of lead exposure on body habitus. Indeed, chronic lead exposure is known to interfere with hypothalamic and pituitary function as reflected by, for example, dopamine concentrations (Kala and Jadhav 1995) and signaling between the hypothalamus and the pituitary gland (Sokol et al. 2002). The hypothalamic–pituitary–adrenal axis, in turn, likely plays a critical role in neuroendocrine regulation of food intake and obesity (Mastorakos and Zapanti 2004). It is possible that lead exposure early in life disrupts this system and results in obesity, an outcome suggested by one previous epidemiologic study in which higher circumpulpal dentine lead levels in shed deciduous teeth were found to prospectively predict, 13 years later, higher body mass index (Kim et al. 1995).

In our study, children from the same immediate family had highly correlated BLLs. Hence, in a given familial residential setting, the finding of lead exposure via a blood lead result in one child may be highly correlated with lead exposures in other siblings. The index exposure child may be a surrogate for overall childhood exposures. Although this finding was based on a limited sample size (*n* = 103), an important implication is also that all children in a family should be tested when one child is found to have a high BLL.

Our study had limitations. We could not determine the contribution of dietary variables (except source of drinking water, which was not significantly associated with BLL) or the presence of environmental sources of lead, because detailed information on these variables was not available from the NFHS. Only information on intake in the preceding 24 hr was provided in broad categories such as intake of fruits, vegetables, other foods, water, and milk. Also, the CDC SD-derived growth reference curves that were used to determine weight/height percentile may not be representative of Indian children. Despite accounting for many factors associated with BLL, our multivariate model had a modest *R*^2^ of 14%. However, this is comparable with other studies looking at determinants of BLL in young children (Ettinger et al. 2004) and adults (Brown et al. 2000; Hu et al. 1996; Lin et al. 2004) (range of *R*^2^ = 8–23%). The lead values in our study were based on field capillary blood testing using a LeadCare analyzer instead of laboratory testing using, for example, atomic absorption spectrophotometry; however, the LeadCare analyzer has been found to be highly accurate and precise in comparison studies (American Academy of Pediatrics Committee on Environmental Health 1998; Parsons et al. 1997), and the NFHS field teams used appropriate skin cleansing techniques to prevent contamination. Another limitation is that BLLs, with a half-life of around 30 days, mostly reflect relatively recent lead exposure, whereas cumulative lead exposure is now appreciated as being the parameter that is probably most predictive of chronic lead toxicity (Hu et al. 1998). However, data for estimating cumulative lead exposure using, for example, an integrated measure of BLLs repeated over time or *in vivo* K-shell X-ray fluorescence measurements of lead in bone (Hu et al. 1998) were not available and, to our knowledge, are not available in any similar type of cohort in India.

Our study found that increasing age, a lower standard of living index, > 95th weight/height percentile, and higher total number of children ever born to the mother were correlated with elevated BLLs in children < 3 years of age in Mumbai and Delhi. Because, lead pollution is widespread in India, the demonstration of factors correlated with BLLs in Indian children may aid in prioritization of children for lead screening and also in studying correctable factors. Although progress has recently been made by phasing out leaded gasoline in India, exposure from many other sources (including the entrainment of lead oxide from combusted leaded gasoline into dust, water, and the food system) is likely to continue for many years to come. Further efforts to control lead pollution, especially in populations at high risk, should be considered.

## Figures and Tables

**Table 1:** Factors associated^a^ with BLLs in 1,081 children < 3 years of age in Mumbai and Delhi, India.

Characteristic	No.	(%)	BLL [μg/dL (mean ± SD)]	β(95% CI)
Age^b^*				
≤3 months	84	7.8	5.8 ± 4.8	Reference
4–11 months	252	23.3	9.6 ± 5.7	3.6 (2.0 to 5.2)
12–23 months	359	33.2	11.8 ± 6.7	6.0 (4.5 to 7.6)
24–35 months	381	35.2	11.8 ± 6.6	5.8 (4.2 to 7.3)
Missing	5	0.5	—	—
Sex^b^				
Female	482	44.6	10.8 ± 6.4	Reference
Male	599	55.4	10.8 ± 6.6	0.5 (−0.3 to 1.3)
Standard of living index^b^*				
High	506	46.8	10.2 ± 6.5	Reference
Intermediate	475	43.9	11.2 ± 6.5	0.7 (−0.2 to 1.6)
Low	49	4.5	13.0 ± 6.7	2.6 (0.7 to 4.6)
Missing	51	4.7	—	—
Mother’s education^b^*				
Higher than secondary	243	22.5	9.8 ± 6.5	Reference
Secondary	411	38.0	10.7 ± 6.3	0.6 (−0.5 to 1.7)
Primary	169	15.6	11.0 ± 7.5	0.9 (−0.4 to 2.3)
No education	258	23.9	11.8 ± 6.1	1.1 (−0.2 to 2.4)
Religion^c^				
Hindu	775	71.7	10.8 ± 6.6	Reference
Muslim	224	20.7	10.8 ± 6.1	−0.2 (−1.2 to 0.8)
Christian	17	1.6	9.5 ± 4.7	−1.7 (−4.8 to 1.3)
Sikh	28	2.6	10.6 ± 6.6	0.3 (−2.2 to 2.7)
Other/no religion	32	3.0	12.0 ± 8.1	1.2 (−1.1 to 3.6)
Missing	5	0.5	—	—
Social class^c^				
Higher caste	694	64.2	10.7 ± 6.3	Reference
Scheduled caste/scheduled tribe^d^	208	19.2	11.3 ± 7.0	0.7 (−0.3 to 1.7)
Other backward caste^d^	179	16.6	10.6 ± 6.7	−0.2 (−1.3 to 0.9)
Birth order^c^*				
One	361	33.4	10.7 ± 7.0	Reference
Two	341	31.5	10.2 ± 6.2	−0.7 (−1.7 to 0.3)
Three	191	17.7	10.9 ± 5.8	−0.2 (−1.3 to 1.0)
≥Four	188	17.4	11.9 ± 6.8	0.8 (−0.5 to 2.0)
Weight/height percentile^c^				
< 5th	219	20.3	10.9 ± 6.6	Reference
5th–95th	741	68.5	10.8 ± 6.5	0.6 (−0.4 to 1.6)
> 95th	34	3.1	12.0 ± 6.8	2.1 (−0.2 to 4.5)
Missing^e^	87	8.1	—	—
Breast-feeding^c^*				
Currently breast-feeding	657	60.8	10.4 ± 6.5	−0.0 (−1.3 to 1.3)
Breast-fed 0–6 months^f^	125	11.6	11.2 ± 7.0	Reference
Breast-fed 7–12 months^f^	136	12.6	11.7 ± 6.7	0.4 (−1.2 to 2.0)
Breast-fed 13–24 months^f^	138	12.8	11.9 ± 6.1	0.2 (−1.5 to 1.8)
Breast-fed 25–35 months^f^	9	0.8	9.6 ± 3.5	−2.5 (−7.0 to 2.0)
Missing	16	1.5	—	—
Anemia^c^				
No anemia (Hb ≥11 g/dL)	310	28.7	10.6 ± 6.8	Reference
Anemia (Hb < 11 g/dL)	768	71.0	10.9 ± 6.4	−0.3 (−1.1 to 0.6)
Missing	3	0.3	—	—
Total children ever born to mother^c^*				
≤2	673	62.3	10.4 ± 6.7	Reference
3–5	358	33.1	11.2 ± 6.2	0.4 (−0.4 to 1.3)
> 5	50	4.6	12.6 ± 6.6	1.4 (−0.5 to 3.3)
BLL (μg/dL)				
< 5	163	15.1	—	—
5–9.9	407	37.7	—	—
10–19.9	414	38.3	—	—
≥20	97	9.0	—	—

Abbreviations: CI, confidence interval; Hb, hemoglobin.

a Differences between categories tested by analysis of variance with log BLL as outcome.

b Regression coefficient (β) reported after including age, sex, standard of living, and mother’s education in the model, with BLL as outcome.

c Regression coefficient (β) reported after including age, sex, standard of living, and mother’s education in the base model, with BLL as outcome, and then including each subsequent variable in the model, one at a time.

d Categories of social class that are distinct but without a hierarchy; both are indicators of lower socioeconomic status in India.

e Includes children coded by NFHS as having implausible values for height and weight.

f Not currently breast-feeding.

* *p* < 0.05.

**Table 2:** Multivariate linear regression predictors of log^a^ BLL in children < 3 years of age in Mumbai and Delhi, India.

	Multivariate regression estimates			
Covariate	Parameter estimate (β)	95% CI	*p*-Value	Relative change (%)^b^
Age (months)				
≤3	Reference	—	—	Reference
4–11	0.61	0.43 to 0.79	< 0.001	84.0
12–23	0.90	0.73 to 1.07	< 0.001	146.0
24–35	0.86	0.68 to 1.03	< 0.001	136.3
Sex				
Female	Reference	—	—	Reference
Male	0.04	−0.04 to 0.12	0.4	4.1
Standard of living index				
High	Reference	—	—	Reference
Intermediate	0.08	−0.01 to 0.18	0.09	8.3
Low	0.28	0.05 to 0.51	0.02	32.3
Mother’s education				
Higher than secondary	Reference	—	—	Reference
Secondary	0.07	−0.05 to 0.18	0.3	7.3
Primary	0.02	−0.13 to 0.18	0.7	2.0
No education	0.09	−0.05 to 0.24	0.2	9.4
Weight/height percentile^c^				
< 5th	Reference	—	—	Reference
5th–95th	0.09	−0.01 to 0.19	0.08	9.4
> 95th	0.27	0.04 to 0.51	0.03	31.0
Total children ever born to mother				
≤2	Reference	—	—	Reference
3–5	0.10	0.01 to 0.19	0.03	10.5
> 5	0.18	−0.02 to 0.39	0.08	19.7
Total model *R*^2^	0.14	—	—	—

Abbreviations: —, missing data; CI, confidence interval; *n* for final multivariate regression model = 945.

a Children with BLLs of 0–0.9 μg/dL were replaced as 0 during log scale conversion.

b Relative percent shift calculated using the formula 100 × (*e*^[β]^ − 1) where β is the parameter estimate.

c Although height/weight percentile was not significant in the univariate analysis, it was significantly correlated with BLL in the multivariate analysis (negative confounding).

**Table 3:** Multivariate regression predictors of elevated^a^ BLLs in children < 3 years of age in Mumbai and Delhi, India.

	BLL ≥10 μg/dL versus < 10 μg/dL
Covariate	Adjusted OR (95% CI)
Age	
≤3 months	Reference
4–11 months	2.8 (1.4 to 5.7)
12–23 months	6.5 (3.3 to 13.0)
24–35 months	5.6 (2.8 to 11.2)
Sex	
Female	Reference
Male	1.1 (0.8 to 1.4)
Standard of living index	
High	Reference
Intermediate	1.6 (1.1 to 2.1)
Low	4.5 (1.9 to 10.4)
Mother’s education	
Higher than secondary	Reference
Secondary	1.4 (1.0 to 2.1)
Primary	1.1 (0.7 to 1.9)
No education	1.6 (1.0 to 2.6)
Weight/height percentile	
< 5	Reference
5–95	1.2 (0.8 to 1.7)
> 95	1.7 (0.8 to 3.8)
Total children ever born to mother	
≤2	Reference
3–5	1.2 (0.9 to 1.7)
> 5	1.5 (0.8 to 3.1)

Abbreviations: CI, confidence interval; OR, odds ratio; *n* for final multivariate regression model = 945.

a Elevated suggests BLL ≥10 μg/dL.
